# An anti-CAPN5 intracellular antibody acts as an inhibitor of CAPN5-mediated neuronal degeneration

**DOI:** 10.18632/oncotarget.22221

**Published:** 2017-11-01

**Authors:** Yan Wang, Xiao Zhang, Zongming Song, Feng Gu

**Affiliations:** ^1^ State Key Laboratory, Key Laboratory of Vision Science, Ministry of Health, Zhejiang Provincial Key Laboratory of Ophthalmology and Optometry, School of Ophthalmology and Optometry, Eye Hospital, Wenzhou Medical University, Wenzhou, Zhejiang 325027, China; ^2^ Henan Eye Institute, Henan Eye Hospital, Henan Provincial People’s Hospital, People’s Hospital of Zhengzhou University, Zhengzhou, Henan 450003, China

**Keywords:** CAPN5, retinal degeneration, photoreceptor, scFv, inhibitor

## Abstract

*CAPN5* has been linked to autosomal dominant neovascular inflammatory vitreoretinopathy (ADNIV). Activation of CAPN5 may increase proteolysis and degradation of a wide range of substrates to induce degeneration in the retina and the nerve system. Thus, we developed an inhibitory intracellular single chain variable fragment (scFv) against CAPN5 as a potential way to rescue degeneration in ADNIV disease or in neuronal degeneration. We report that overexpression CAPN5 increases the levels of the auto-inflammatory factors toll like receptor 4 (TLR4), interleukin 1 alpha (IL1alpha), tumor necrosis factor alpha (TNFalpha) and activated caspase 3 in 661W photoreceptor-like cells and SHSY5Y neuronal-like cells. Both C4 and C8 scFvs specifically recognize human/mouse CAPN5 in 661W cells and SHSY5Y cells, moreover, both the C4 and C8 scFvs protected cells from CAPN5-induced apoptosis by reducing the levels of activated caspase 3 and caspase 9. The cellular expression C4 scFv reduced levels of the pro-inflammatory factor IL1-alpha activated caspase 3 in cells after CAPN5 overexpression. We suggest that CAPN5 expression has important functional consequences in auto-inflammatory processes, and apoptosis in photoreceptor like cells and neural-like cells. Importantly, the specific intracellular targeting of antibody fragments blocking activation of CAPN5 act as inhibitors of CAPN5 functions in neural like cells, thus, our data provides a novel potential tool for therapy in CAPN5-mediated ADNIV or neurodegenerative diseases.

## INTRODUCTION

*CAPN5* encodes calpain-5, a member of the calcium-activated cysteine protease family [1, 2]. CAPN5 has been associated with autosomal dominant neovascular inflammatory vitreoretinopathy (ADNIV) [3–6], obesity [7], Huntington’s disease [8, 9], and polycystic ovary syndrome [10]. CAPN5 has been found to be localized in the cytoplasm and nucleus of photoreceptor cells, neuronal cells in the retina, and also in the central nervous system [11, 12]. The members of the calpain family usually show elevated proteolytic functions in nervous system diseases. Calpain is a ubiquitous calcium-sensitive protease that is essential for normal physiologic neuronal function [13]. However, alterations in calcium homeostasis lead to persistent, pathologic activation of calpain in a number of neurodegenerative diseases [14]. Pathologic activation of calpain induces the cleavage of substrates that negatively affect neuronal structure and function, leading to inhibition of essential neuronal survival mechanisms [15]. Thus, Inhibition of activated calpain represents an ideal therapeutic strategy in brain injury [16–18], Alzheimer’s disease [19], Parkinson’s disease [20], Huntington’s disease [8], multiple sclerosis [21], optic injury [22], as well as retinal degenerative diseases [23]. The C. elegans ortholog of CAPN5, TRA-3, has essential regulated functions for necrotic neuronal death [24, 25]. Autosomal dominant neovascular inflammatory vitreoretinopathy (ADNIV) is an inherited autoimmune uveitis and vitreoretinal degeneration [26]. ADNIV is caused by mutations of the *CAPN5* gene which leads to photoreceptor degeneration, autoimmune uveitis, and retinal neovascularization. It has been found that mutations of *CAPN5* activated CAPN5 protein that generates the various pathological features involved in blindness and could be therapeutically relevant [27, 28].

Because activating mutations of CAPN5 play pivotal roles and have a significant effect on degeneration of photoreceptor cells at an early stage in human ADNIV patients [3–6], we generated intracellularly expressed single chain antibody fragments against CAPN5 to block possible active-CAPN5 substrate-mediated cell damage including apoptosis, autoimmune-activation, and retinal photoreceptor cell degeneration. This may be a possible way to treat of activated-CAPN5 induced photoreceptor cell and neuronal cell degeneration in ADNIV and neurodegenerative diseases.

## RESULTS

### Overexpression of CAPN5 induces apoptosis and expression of pro-inflammatory factors in neuronal cells

**Figure 1:**
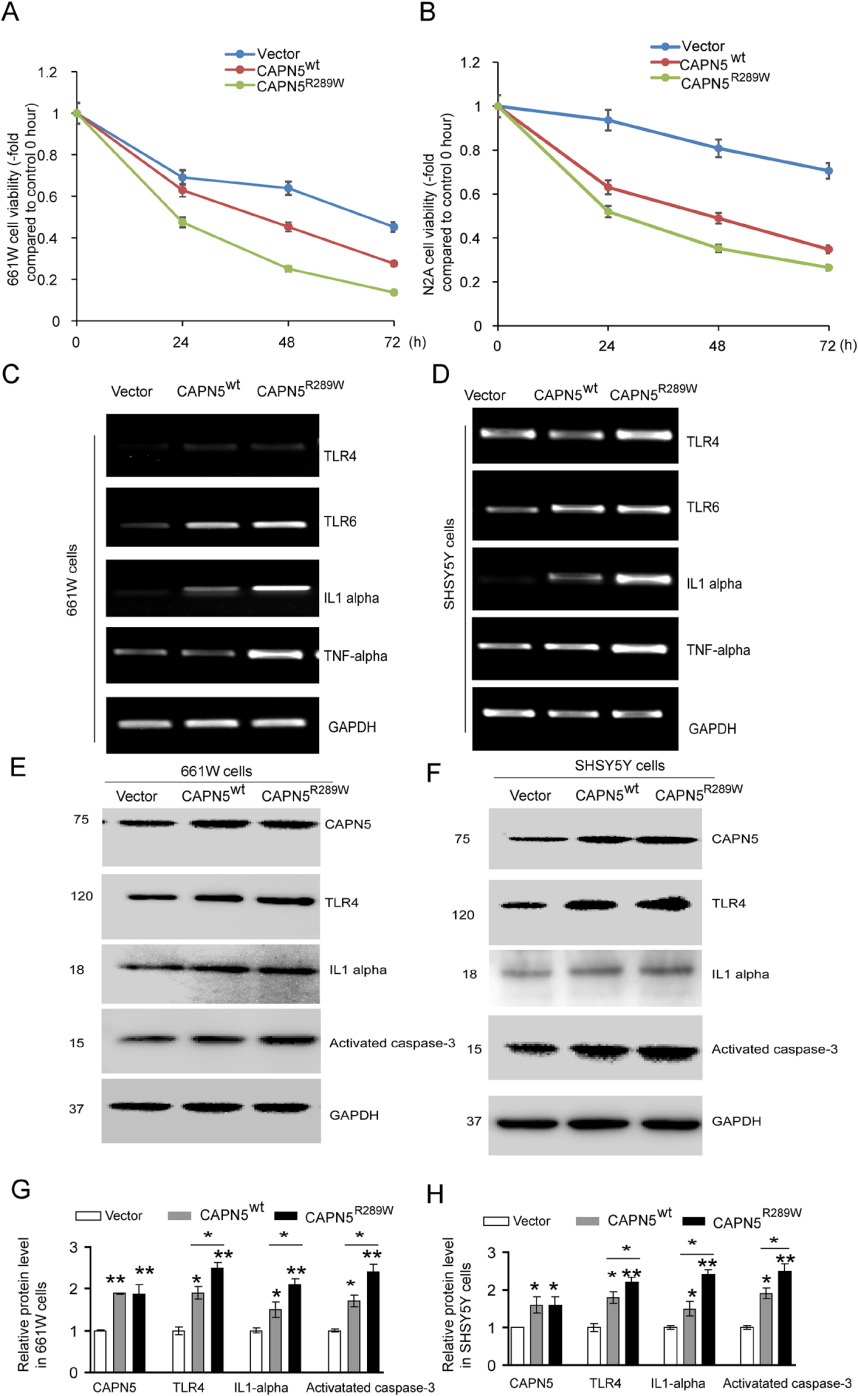
Overexpression CAPN5 induced decreasing viability of neural-like cells, increased expression levels of TLR4, IL1alpha and caspase 3 Photoreceptor-like 661W cells and neural-like N2A cells or SHSY5Y cells were transfected with CAPN5^wt^ and CAPN5^R289W^ plasmids respectively, at indicated hours post-transfection. **(A)** Cell viability of 661W cells were measured by MTT assay and calculated. **(B)** The related viability of N2A cells. After sixty-hour transfections, the 661W cells and SHSY5Y cells were lysed for immunoblot and RT-PCR analysis, respectively. mRNA or protein levels of CAPN5, TLR4, TLR6, IL1alpha, TNFalpha and GAPDH were determined. **(C)** RT-PCR measurements for 661W cells. **(D)** RT-PCR measurements for SH-SY5Y cells. Protein levels of CAPN5, TLR4, IL1alpha, and activated caspase 3 were measured by immunoblotting. **(E)** Representative images for 661W cells. **(F)** Representative images for SHSY5Y cells. **(G)** The density of bands from (E), and **(H)** the density of bands from (F) were measured and normalized to GAPDH by Image J and calculated. Values represent mean ± SEM of density compared with empty vector controls from triplicate experiments. *, *p*<*0.05*, **, *p*<*0.01*, Kruskal–Wallis test was used.

It has been shown that CAPN5 activation may induce degeneration of photoreceptor cells in the eye and neuronal cell death in the nerve system [6, 9]. To characterize the roles of *CAPN5* in photoreceptor cells and neuronal-like cells, we transfected plasmids (CAPN5^wt^ and CAPN5^R289W^) into 661W cells, N2A cells and SHSY5Y cells, respectively. After 24, 48, 72 hours transfections in 661W and N2A cells, the cell viability of 661W and N2A were both strongly reduced by CAPN5 and CAPN5 R289W overexpression in a time-transfection dependent manner (Figure 1A, 1B). Moreover, The CAPN5 mutant R289W overexpression decreased the more viability of cells when compared to CAPN5 wt transfections in both 661W and N2A cell lines. After 60 hours post-transfection, both the CAPN5 and CAPN5 mutant R289W vectors transfection increased the mRNA levels of TLR4/6, IL1alpha and TNFalpha when compared to empty vector transfection, and this was especially pronounced for the mutant CAPN5 R289W expression which increased both caspase 3 activation and IL1alpha levels when compared to CAPN5 wt transfection in both 661W and SHSY5Y Cell lines (Figure 1C, 1D). After 60 hours transfections, we also detected the protein levels of TLR4 (*p*<*0.05*,-1.6 folds in CAPN5 wt; *p*<*0.01*, -2.3 folds in CAPN5 R289W in 661W cells. *p*<*0.05*, -1.7 folds, in CAPN5wt; -2.2 folds in CAPN5 R289W in SHSY5Y cells), IL1 alpha (*p*<*0.05*, -1.4 folds in CAPN5 wt; *p*<*0.001*, -2.0 folds in CAPN5 R289W in 661W cells. *p*<*0.05*, -1.5 folds in CAPN5wt; *p*<*0.01*, -2.4 folds in CAPN5 R289W in SHSY5Y cells), TNF alpha (*p*<*0.05*, -1.5 folds in CAPN5 wt; *p*<*0.01*,-2.2 folds in CAPN5 R289W in 661W cells.*p*<*0.05*, -1.8 folds in CAPN5 wt, *p*<*0.01*, -2.2 folds in CAPN5 R289W in SHSY5Y cells) were increased by CAPN5 overexpressions (*p<0.001*, -1.8 folds in 661W cells; *p*<*0.01*, -1.6 folds in SHSY5Y cells). Moreover, expression of CAPN5 R289W led to higher protein levels of TLR4, IL1 alpha and activated caspase 3 in both cell lines (Figure 1E, 1F, 1G, 1H). These data indicate that CAPN5 exerts its effects on TLR4/IL1/TNF alpha expression at the transcriptional level, and overexpression of CAPN5 increased caspase 3 activation and expression of pro-inflammatory proteins as TLR4, IL1alpha and TNF alpha. It is interesting that the mutant CAPN5 R289W shows the stronger effects on caspase 3 activation and protein levels of the TLR4 pathway when compared to wild-type CAPN5 overexpression in neuronal cells. We also have been found that overexpression of CAPN5 vectors shortened neurite lengths in mouse neuroblastoma N2A cells when compared to empty vector control transfection (data not shown). Together, these results suggest that overexpression CAPN5 induces activation of auto-inflammation and apoptosis in neural-like cells.

### Screening and selection of specific CAPN5 scFvs by phage display

**Figure 2:**
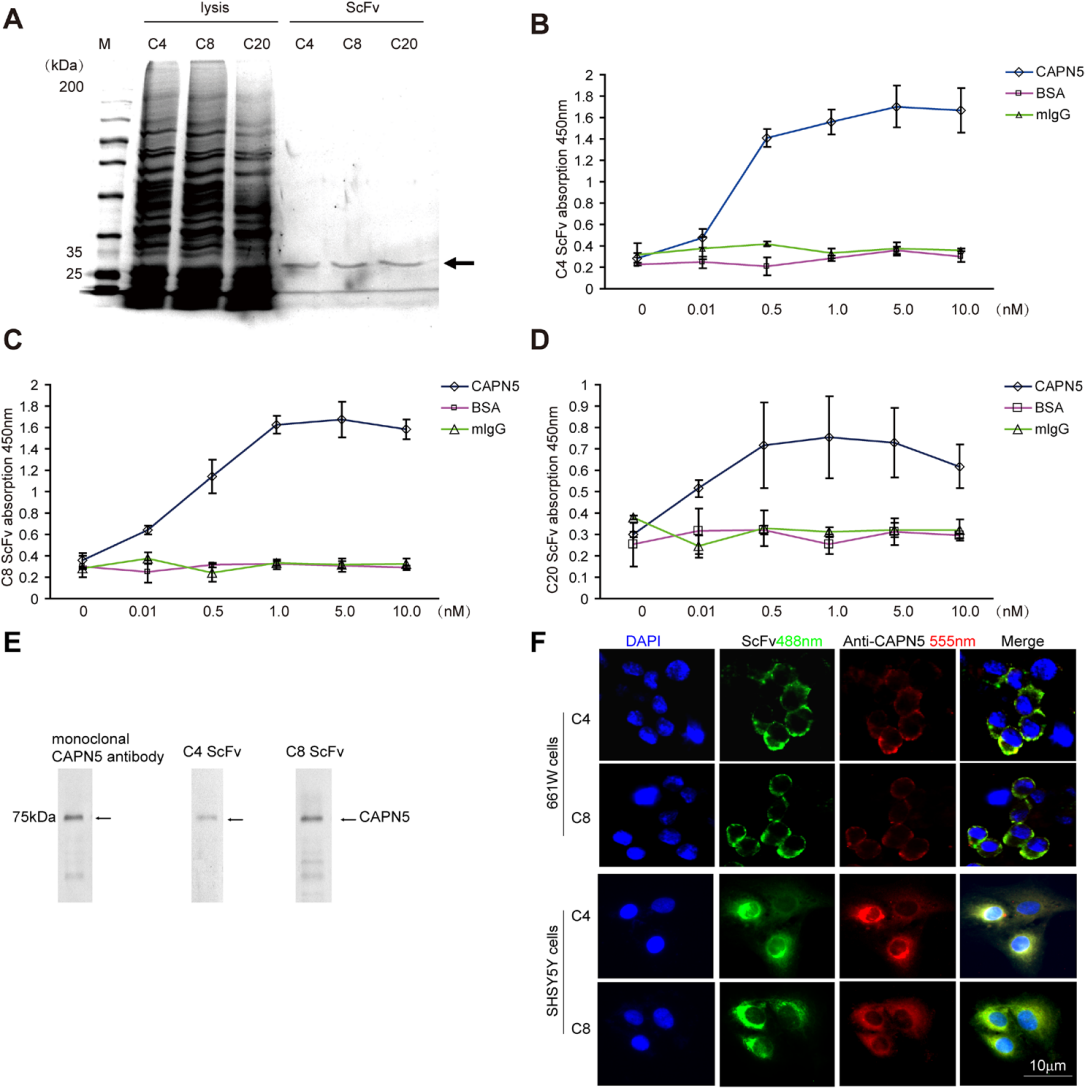
Selection and binding characteristics of CAPN5 scFvs in 661W cells and SHSY5Y cells Selected C4, C8, C20, scFv-phages were infected into HB2151 strain *E. coli.* cells and induced by IPTG overnight, purified and detected by SDS-PAGE respectively. **(A)** Purification of scFvs. The arrow denotes that the approximate molecular weight of scFvs at 30kDa. **(B)** Values represent mean±SEM OD_450nm_ for binding of C4 scFv to recombinant CAPN5, normal mouse IgG, and BSA proteins from three independent experiments. The ninety-six well plates were coated with recombinant CAPN5, normal mouse IgG and BSA at the indicated concentrations, and binding capability of scFvs was detected by an anti-c-myc monoclonal antibody followed by goat anti-mouse HRP secondary antibody with ELISA. **(C)** Binding capability of C8 scFv to indicated recombinant proteins. **(D)** C20 scFv binding characteristics measured by ELISA. **(E)** 661W cells were lysed and immunoblotted by anti-CAPN5 C4/C8 scFv and monoclonal anti-CAPN5 antibody respectively. The arrows denote the specific molecular weight of CAPN5 at 75 kDa. **(F)** C4 and C8 scFvs bound to living 661W cells and SH-SY5Y cells. C4 or C8 ScFvs (green) and goat anti-CAPN5 (red) were incubated with living 661W cells and SH-SY5Y cells at 10μg/ml in PBS, for 1 h, cells were then fixed and detected by immunofluorescence. Bar 10μm is shown in the lower photo for all panels.

Activating CAPN5 mutations have been shown accelerate the degeneration of photoreceptor cells, auto-immunity inflammatory in retinal cells, and neuronal cell death in the nervous system [27, 28]. To design an inhibitor of CAPN5 to protect cells, we used phage display to screen scFvs against human CAPN5. We purified and selected three scFvs we called C4 and C8, C20 (Figure 2A) binds to the purified CAPN5 by direct ELISA. Both C4 and C8 scFvs did not bind to normal mouse IgG or BSA (Figure 2B, 2C). We detected C20 scFv non-specific binding characteristics by ELISA (Figure 2D). To confirm whether C4 and C8 scFvs antibodies reacted with wild type CAPN5 protein in cells, we incubated C4 scFv and C8 scFv with total proteins from 661W cell lysates which separated and transferred onto PVDF membrane, then incubated membrane with anti-His tag and detected the signals by secondary antibody. In immunoblot assay, we found the specific 75 kDa band was detected by both C4 and C8 scFvs, the bands at 75 kDa were seem as the band detected by a commercial anti-CAPN5 antibody (Figure 2E). We also pre-incubated the living cells with C4/C8 scFvs for 2 hours, then fixed cells, incubated cells with anti-His tag/ a commercial goat anti-CAPN5 antibody respectively, following with secondary antibodies. We found that scFvs bind to cytoplasmic CAPN5, the immunofluorescence signal co-localized with signals detected by the commercial antibody against CAPN5 in 661W cells and human SHSY5Y cells (Figure 2F). These data suggest that we successfully produced specific scFvs against human CAPN5 by phage display, and moreover, the antibody fragments also react with mouse CAPN5 in *vitro*. Both C4 scFv and C8 scFv could be entered into the human living SHSY5Y cells and mouse living 661W cells.

### CAPN5 scFvs protect 661W cells and SHSY5Y cells from H_2_O_2_ and CAPN5 induced apoptosis

**Figure 3:**
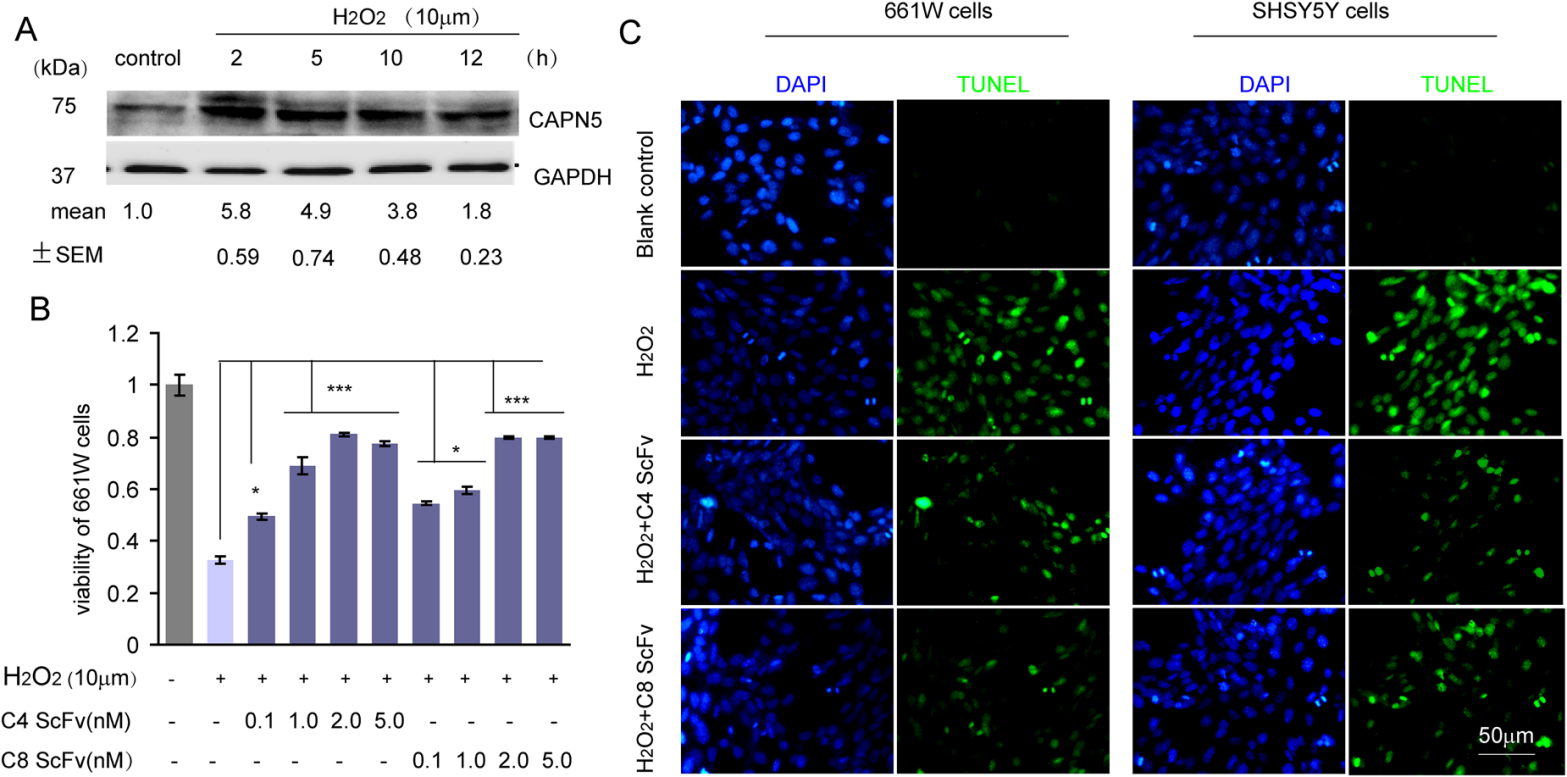
C4 and C8 scFvs rescued H_2_O_2_-induced cell apoptosis of 661W and SHSY5Y cells **(A)** 5×10^6^ 661W cells were treated with 10μM H_2_O_2_ for the indicated times with serum free medium in six well plates, and cells were lysed for immunoblot assay. Protein levels of CAPN5 and GAPDH were detected and C4 and C8 scFvs are shown. The densitometric values for levels of CAPN5 were measured and shown under each band compared with density of GAPDH. **(B)** 3×10^3^ 661W cells were treated with H_2_O_2_ at 10μM for 2 hours with continued culture in serum free medium in the presence of C4 and C8 ScFvs for 24 h in 96 well plates. Cell viability was assessed by the MTT assay, and absorbance was read at 450 nm. The values represent mean±SEM of relative cell viability in ScFvs treatment groups in the absence or presence of H_2_O_2_ compared with controls. * *p*<0.05, **p<0.01. One way ANOVA and Tukey’s post hoc test were used for statistical analysis. **(C)** 4×10^4^ 661W cells or SHSY5Y cells were plated onto cover-slips and treated with H_2_O_2_ for 2 hours in continuous culture in serum free medium in the presence of C4 or C8 scFvs. The cells were fixed for TUNEL staining and DAPI for nuclear staining. Representative images show the positive TUNEL staining (Green) with DAPI positive staining (Blue) cells at 400× magnification.

**Figure 4:**
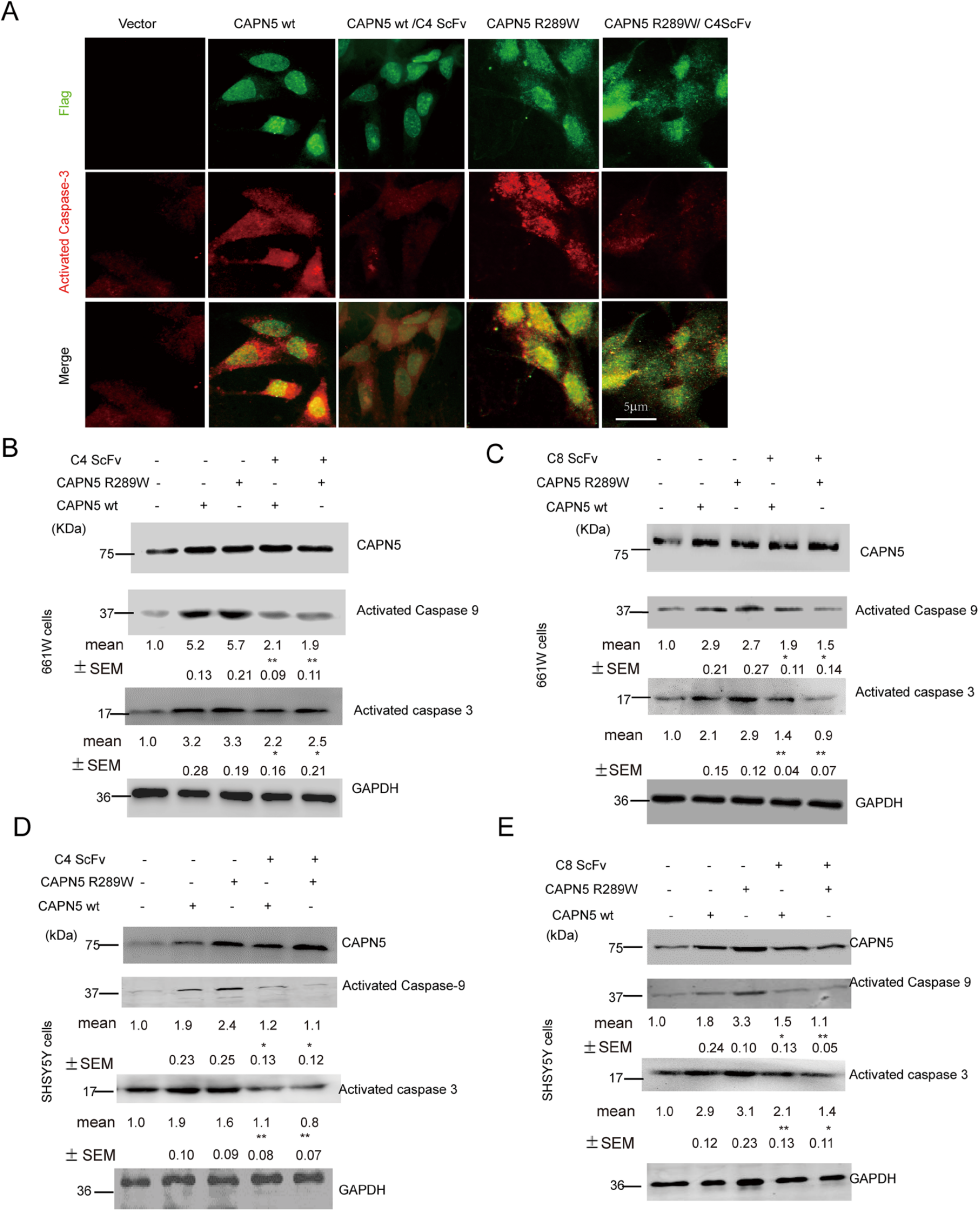
C4 and C8 scFvs reduced activated caspase 3/9 levels induced by CAPN5 overexpression in 661W and SHSY5Y cells 5×10^6^ 661W cells and 3×10^6^ SHSY5Y cells were transfected with CAPN5 plasmids and empty vectors, and 60 hours post-transfection, the cells were treated continuously with purified C4 scFv or C8 scFv at 10μg/ml. Levels of activated caspase 3/9, and CAPN5 were detected by immunofluorescence and immunoblot respectively. **(A)** ScFvs reduced immunofluorescence of activated caspase 3 in 661W cells after CAPN5/Flag vector transfection. Cells were fixed and stained with anti-Flag followed by green secondary donkey anti-mouse Alexa 488nm fluorescence antibody; Anti-CAPN5 with red secondary donkey anti-goat Alexa 544nm-labeled antibody. Bar, 5 μm in upper panel for all panels in (A). **(B)** C4 scFv against CAPN5 decreased the levels of activated caspase 3/9 in 661W cells. **(C)** C8 scFv against CAPN5 decreased the levels of activated caspase 3/9 in 661W cells. **(D)** C4 scFv against CAPN5 decreased the levels of activated caspase 3/9 in SHSY5Y cells. **(E)** C8 scFv against CAPN5 decreased the levels of activated caspase 3/9 in SHSY5Y cells. GAPDH was used as a loading control. The protein levels of CAPN5 and caspase 3/9 were detected, measured and normalized to GAPDH by Image J software. The values presents as mean±SEM shown shown under each band from triplicate experiments compared with cells in the absence of ScFv treatments. * *p*<0.05, **p<0.01. One way ANOVA with Tukey’s post hoc test was used for statistical analysis.

To analyze whether the scFvs against CAPN5 could protect cells from CAPN5-induced apoptosis, we used H_2_O_2_ to induce the death of 661W cells. We found that the protein levels of CAPN5 were dramatically increased by H_2_O_2_ treatment. After 2 hours exposure to H_2_O_2_, the viability of 661W cells was enhanced by 24 hours ScFv pre-treatment at the indicated concentrations when compared to H_2_O_2_ exposure in the control group (Figure 3A, 3B). We also found that the C4 scFv significantly reduced the numbers of TUNEL-positive 661W (-32.1% of control group) and SHSY5Y cells (-41% of control group) compared with H_2_O_2_ exposed controls (-78.9% in 661 cells, -87.3% in SHSY5Y cells) (Figure 3C). These data suggested that CAPN5 scFvs protect cells from H_2_O_2_-induced apoptosis, and neutralize the functions of CAPN5 in response to H_2_O_2_ treatment. To further determine whether CAPN5 ScFvs act as inhibitors of CAPN5 induced apoptosis, we added scFvs alongside overexpression of CAPN5 in 661W cells and SHSY5Y cells. After 48 hours of CAPN5 overexpression, scFvs were added to the culture medium. Activated caspase 3 was measured by immunoflourescence (Figure 4A) and caspase 9 levels were decreased by C4 and C8 scFv treatments when compared to controls. Similar trends were detected in 661W cells (Figure 4B, 4C) and SHSY5Y cells (Figure 4D, 4E). These data confirm that scFvs rescue the apoptosis of neuronal cells by targeting CAPN5.

### Generation of intracellular antibody fragments against CAPN5

**Figure 5:**
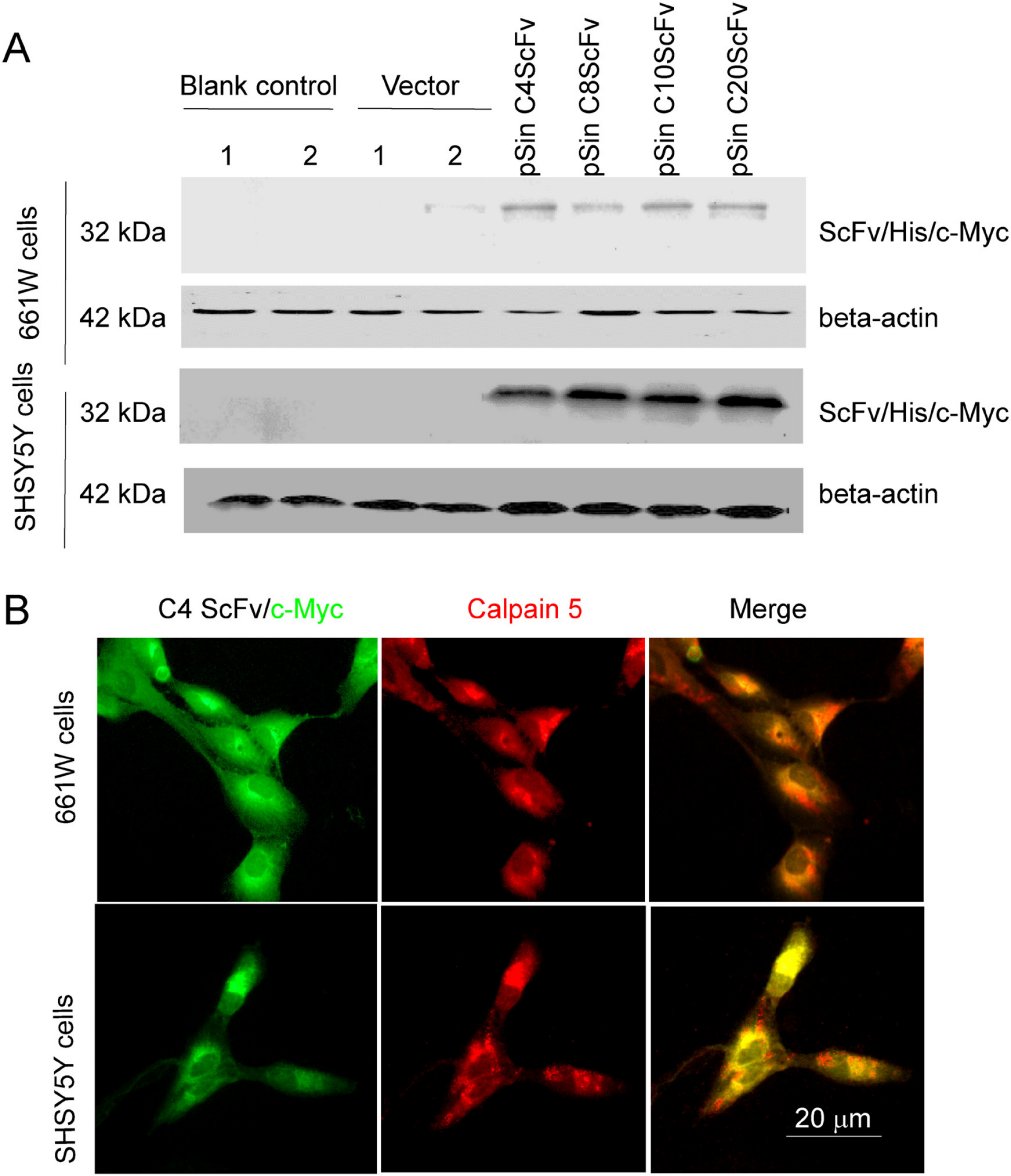
Generation of intracellular CAPN5 antibody fragments in 661W cells and SHSY5Y cells C4 or C8, C10, or C20 scFvs were sub-cloned into the pSin vector and then transfected into 661W cells or SH-SY5Y cells. After 60 hours post-transfection, the intracellular scFvs were detected by immunofluorescence or immunoblot. **(A)** Expression levels of scFvs in 661W cells. Vector control was empty vector, blank control was cells in the absence of transfection. 1 and 2 denote duplicate samples. GAPDH was used as a loading control. **(B)** Immunofluorescence of C4 scFvs colocalized with CAPN5 in 661W cells and SHSY5Y cells. His/myc tagged C4 scFv was detected by anti-c-myc 9E10 antibody and secondary green 488nm donkey anti-mouse antibody. Endogenous CAPN5 was detected by goat anti-CAPN5 antibody followed by with red secondary Alexafluor 555nm antibody. Images were taken at 400×magnification.

To generate stable and durable intracellular CAPN5 scFvs in cells we constructed four pSin scFv plasmids. After seventy-two hours post-transfection with pSin C4ScFv, pSin C8ScFv, pSin C10ScFv, and pSin C20ScFv detected all scFv proteins highly expressed in 661W cells and SHSY5Y cell lysates, in an immunoblot assay (Figure 5A). We also selected and transfected the pSin C4scFv into 661W cells and SHSY5Y cells, after forty-eight hours post-transfection, we found co-localization of intracellular C4 ScFv with endogenous CAPN5 in 661W cells and SHSY5Y cells by immunofluorescence (Figure 5B). These data suggest that we successfully expressed intracellular specific C4 scFv antibody directly against endogenous CAPN5 in 661W photoreceptor like cell and SHSY5Y neural-like cells.

### Intracellular antibody against CAPN5 decreased the levels of IL1alpha and activated caspase-3 in cells with CAPN5 overexpression

**Figure 6:**
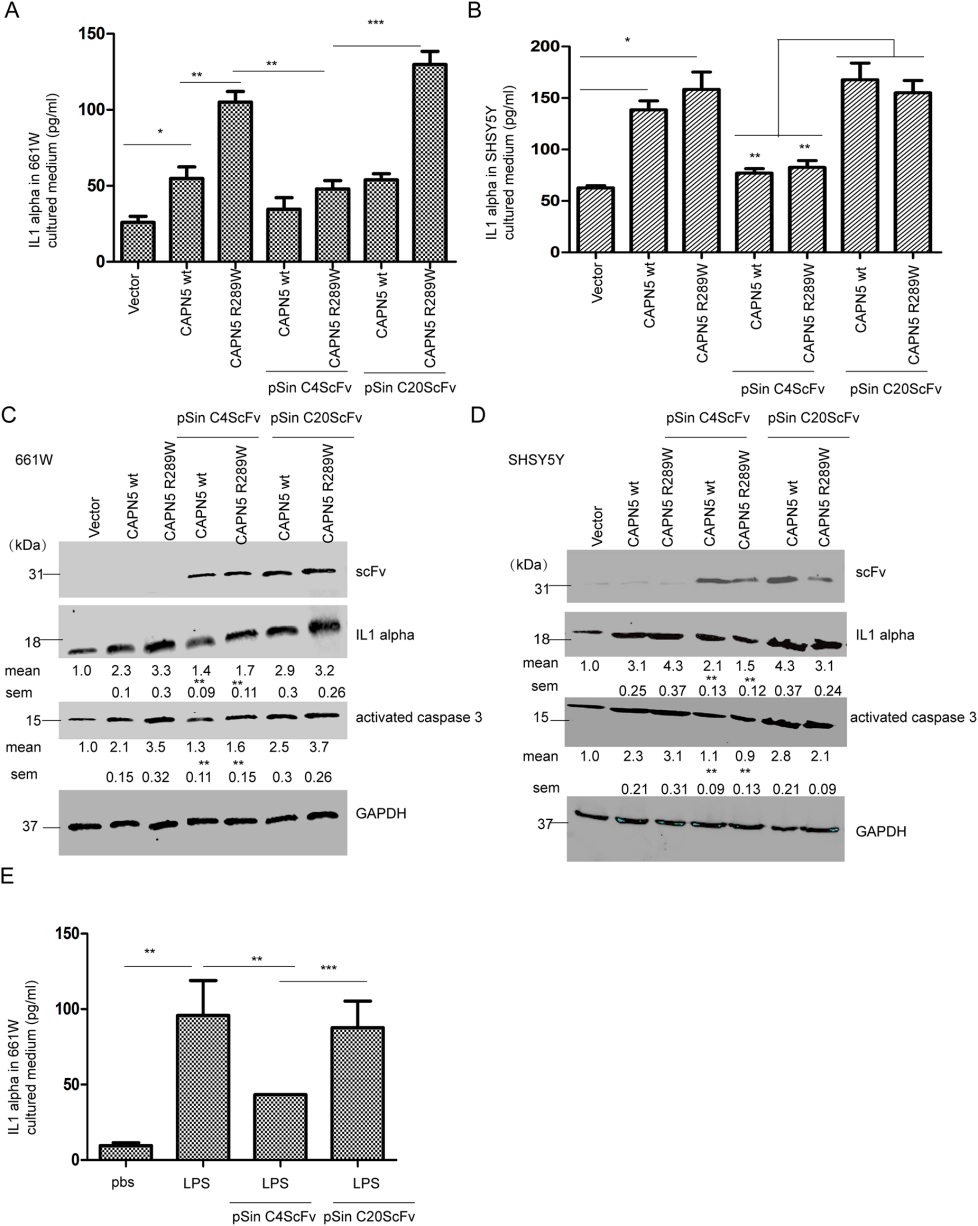
Intracellular CAPN5 antibody fragment inhibited secretion of IL1alpha, and caspase 3 activation induced by overexpression CAPN5 and LPS 3.0×10^6^ 661W cells and 4.0×10^6^ SH-SY5Y cells were treated with LPS at 100 ng/ml with serum free medium, then transfected with CAPN5 plasmids, empty vectors, or co-transfected with CAPN5 plasmids and C4 scFv plasmid or C20 scFv plasmid, respectively. After 60 hours post-transfection, culture medium was collected and IL1alpha detected by ELISA. **(A)** Secretion of IL1alpha in 661W cells. **(B)** Secretion of IL1alpha in SH-SY5Y cells.*, *p*<*0.05*, **, *p*<*0.01*, ***, *p<0.001*, Kruskal–Wallis test was used. After 60 hours post-transfection, cell lysates were detected by immunoblotting. **(C)** The protein levels of IL1alpha and activated caspase 3 were inhibited in 661W cells with CAPN5 transfections by intracellular scFv transfection. **(D)** Protein levels of IL1alpha and activated caspase 3 were inhibited in 661W cells with CAPN5 transfection by intracellular scFv transfection. The protein levels of IL1alpha was detected by specific antibodies. GAPDH was used as a loading control. The protein levels of IL1alpha and activated caspase 3 were detected and measured by Image J software. The values are presented as mean±SEM shown under each band from triplicate experiments compared with cells with CAPN5 vector transfections and by omitting scFv treatments in (C) and (D). * *p*<0.05, **p<0.01, ***, *p*<0.001. One way ANOVA with Tukey’s post hoc test was used for statistical analysis. 3.0×10^6^ 661W cells were treated with LPS at 200ng/ml for 24 h, then transfected with pSin C4 scFv or pSin C20 scFv for 48 h, cells cultured medium was collected and detected by ELISA. **(E)** Intracellular C4 scFv decreased the secreted level of IL1alpha in 661W cells after LPS treatment. The values are presented as mean±SEM shown in graphs from triplicate experiments compared with cells with CAPN5 vector transfections and omitting scFv treatments or C20 scFv plasmid transfection in (A), (B), (E). * *p*<0.05, **p<0.01, ***, *p*<0.001. One way ANOVA with Tukey’s post hoc test was used for statistical analysis.

To confirm whether the intracellular antibody against CAPN5 could block CAPN5-mediated inflammation and apoptosis, we co-transfected CAPN5 plasmids with pSin scFv plasmids into 661W and SHSY5Y cells, respectively. Sixty hours post-transfection, the secretion of IL1alpha in cultured medium as measured by ELISA was increased after CAPN5wt plasmid (Kruskal–Wallis test, *p<0.001*, 51±6.7 pg/ml vs 23±5.14 pg/ml control in 661W cells; *p*<*0.05*, 135±16.7 pg/ml vs 53±7.15 pg/ml control in SHSY5Y cells) and CAPN5 mutant R289W transfection (Kruskal–Wallis test, *p*<*0.001*, 102±11.4 pg/ml vs control in 661W cells; 161±21.31 pg/ml vs control in SHSY5Y cells), while the levels of secreted IL1alpha (Kruskal–Wallis test, *p*<*0.01*, 661W cells, 37±2.13 pg/ml in CAPN5wt; 43±3.18 pg/ml in CAPN5 R289W; *p*<*0.01*, SHSY5Y cells, 69±2.56 pg/ml in CAPN5 wt, 73±4.18 pg/ml in CAPN5 R289W) were reduced after co-transfection of CAPN5 plasmids with pSin C4 scFv plasmid in both cell lines (Figure 6A, 6B). The expression levels of IL1alpha and activated caspase-3 were also decreased in co-transfected cells compared with CAPN5 plasmid transfection alone (Figure 6C, 6D). The selected C20 ScFv did not specifically recognize CAPN5, and was used as a negative control (Figure 6). This did not alter the levels of IL1alpha and activated caspase-3 in cells with CAPN5 transfection. These data thus suggest that the intracellular C4scFv specific against CAPN5, blocked CAPN5-induced secretion of IL1alpha, as well as protein levels of IL1alpha, and activated caspase 3 in 661W photoreceptor like cells and SHSY5Y neural-like cells. Previously, we have been found LPS induced highly expression of CAPN5 and IL1 alpha in 661W cells (data not shown). Here, we also found that intracellular C4 scFv inhibited IL1alpha secretion from LPS-stimulated 661W cells (Figure 6E). Taken together, these data demonstrate that the CAPN5-specific intracellular antibody inhibited CAPN5 functions when CAPN5 was overexpressed in neuronal cells or after LPS induced inflammation.

## DISCUSSION

In this study we initially showed that overexpression of CAPN5 decreased viability of neural-like cells, the expression levels of TLR4 and caspase 3 activation were enhanced by CAPN5 overexpression, showing that CAPN5 is either directly or indirectly involved in increased levels of TLR4 and activated caspase 3. Moreover, we also saw increased levels of IL1alpha which is downstream of TLR4 signaling, as a downstream mechanism of autoimmunity pro-inflammation [31]. The enhanced secretion of IL1alpha, and caspase 3 activation result in degeneration of photoreceptor-like cells and neural-like cells by CAPN5 overexpression. Interesting, overexpression of the mutant CAPN5 R289W was decreased viability of neural-like cells and enhanced the higher levels of caspase 3/9 and TLR4/IL1alpha when compared with wild type CAPN5 overexpression. In agreement with our findings, activated CAPN5 induced increased inflammatory factors through TLR4/6 autoimmunity inflammation pathways in retinal degeneration in ADNIV patients [5, 6]. Previously, we have observed a novel CAPN5 R289W mutant affect ADNIV patients in a Chinese family. We speculated this mutant stabilized catalytic core domain II and enhanced CAPN5 catalytic activation in construct conformation. There is also an evidence have been reported that the mutant R243L increased activation of CAPN5 catalytic core domain to stimulate TLR4 autoimmunity inflammation in *CAPN5*^*R243L*^ transgenic mice retina [28]. Thus, we considered that the mutant CAPN5 R289W increased degeneration of photoreceptor cells by activated caspase 3 and TLR4 auto-inflammatory pathway.

It also has been found that in neurodegenerative Huntington’s disease, CAPN5 is abnormally activated regulator for proteolytic of htt protein in neural cell death [8]. The evidence presented here suggests that activation of CAPN5 leads to neural degeneration by increased activated caspase 3/9 in cells. Thus, we screened and selected specific scFvs which block CAPN5, we successfully constructed intracellular antibody fragment expression plasmids to express specific scFvs against endogenous CAPN5 in cells. The CAPN5 intracellular antibody fragments inhibited secretion of IL1alpha induced by LPS or CAPN5 overexpression. The intracellular antibody fragment neutralized activation of CAPN5 by overexpression in neural-like cells and protected cells from activated CAPN5-incuded pro-inflammation and cell apoptosis. Taken together, these findings strongly suggest that CAPN5 activation causes retinal degeneration and is also involved in neuronal degeneration.

CAPN5 is a member of the calpain family, but lacks the EF calcium binding domain, it may has similar characteristics and substrates as others calpains, like classical calpain1/2 in the nerve system and retina [8]. Although calpains have vast numbers of proteases and proteolytic complexes in nerve systems, calpains are very few that are directly enzymatic activated dependent by Ca^2+^ in signal transduction. In addition, calpains are modulator proteases that perform proteolysis to modulate rather than abolish the function of their substrate [32]. Since, the Ca^2+^ signaling and proteolysis of CAPN5 need to be further explored to fit CAPN5 into our subject of interest. Moreover, in his study, the precise scFv binding/blocking domain(s)/epitopes of CAPN5 were still unknown. We have been found purified CAPN5 activity by cleavage of a common calpains’ substrate AC-LLY-AFC dependent Ca^2+^ concentration. The C4 scFv antibody inhibited CAPN5 activity of cleavage for AC-LLY-AFC (data not shown). Therefore, the screening of scFv against the specific catalytic domain of CAPN5 and substrates of CAPN5 could be strategies to investigate molecular mechanism underlying its related neuronal degenerative diseases.

Recently, many inhibitors against calpain 1/2 have been generated to inhibit neuronal degeneration and ophthalmic diseases *in vitro* and *in vivo* [32–34]. It has been report that calpain-1 is hyperactivated in the AD brain [35], and calpain inhibitors can improve memory and synaptic function in mice APP overexpressing AD model [36]. Calpain 1/2 inhibitors, ALLNal and SNJ1945 are therapeutically beneficial in LIS1-related lissencephaly [37]. The inhibition of calpain 2 is also benefit to relieving photoreceptor degeneration in retinitis pigmentosa [38]. However, these inhibitors were still not sufficiently specific to distinguish calpains from other proteases in almost all cells. Thus, we firstly screened and generated the specific intracellular anti-CAPN5 scFv to inhibit CAPN5 overexpressing in photoreceptor cells and neuronal like cells. ScFv offer small size and low immunogenicity and can be used in gene delivery system to target proteins and neutralized harmful protein activations [32]. The intracellular expressed and targeting antibody (intrabody) has been used in therapeutically aims in neurodegenerative diseases [39]. Here, we generated pSin vector encoding scFv to target intracellular CAPN5 protein, and block CAPN5-induced inflammation and apoptosis in neuronal degenerative processes. We could also be further exploring adeno associated virus (AAV) delivery CAPN5 scFv as a treatment strategy for *CAPN5* mutations-linked ADNIV in eye and activated CAPN5 related-neurodegenerative diseases in central nerve system.

In summary, our observations support the view that activated CAPN5 induced activation of TLR4 and caspase 3 pathways in auto-inflammation and apoptosis, thereby leading to degeneration of photoreceptors and neural degeneration. We developed intracellular antibody fragments against cellular CAPN5 and inhibited the process of auto-inflammation and cell death by activated CAPN5 induced. These potential intracellular antibody fragments could be further used for therapy in ADNIV mice model and neurodegenerative diseases.

## MATERIALS AND METHODS

### Antibodies and reagents

Antibodies against the following proteins were used in this study (listed in Table 1): glyceraldehyde-3-phosphate dehydrogenase (GAPDH), mouse/human calpain-5 (CAPN5), c-myc tag, His-tag, mouse/human TLR4 and IL1alpha, mouse/human activated caspase 3/9. Secondary antibodies coupled to horseradish peroxidase (HRP) or fluorescein were also obtained (Table 1). Highly purified normal human/mouse immunoglobulin (IgG) and bovine serum protein were obtained from Sino Biological Inc. (Beijing, China). MTT (3, 4, 5-dimethylthiazol-2-yl)-2, 5-diphenyltetrazoliumbromide) was used to determine cell viability (R&D Systems). DAPI (4’, 6-diamidino-2-phenylindole) was used to stain cell nuclei. IPTG (isopropyl β-D-1-thiogalactopyranoside) was used to induce expression of protein (Beyotime). Poly-D-lysine (PDL) (Beyotime) was used as a substrate for cell culture. Lipopolysaccharide (LPS, extract from *Salmonella enterica* serotype enteritidis) (Sigma-Aldrich, Shanghai, China) was used to induce inflammation in cells.

**Table 1:** Antibodies used in this study

Antibody	Supplier	Dilution
Monoclonal anti-CAPN5	Santa Cruz Biotechnology, Santa Cruz, CA, USA	1:500
Goat anti-CAPN5	Santa Cruz Biotechnology	1:700
Monoclonal anti-C-myc tag (9E10)	Santa Cruz Biotechnology	1:500
Monoclonal anti-GAPDH	Beyotime Biotechnology	1:800
Monoclonal anti-activated caspase 3	Beyotime Biotechnology	1:500
Monoclonal anti-His tag	Beyotime Biotechnology	1:400
Rabbit anti-TLR4	Boshide Biotechnology, Wuhan, China	1:400
Monoclonal anti-IL1alpha	Boshide Biotechnology	1:400
Rabbit anti-goat IgG HRP	Beyotime Biotechnology	1:1000
Rabbit anti-mouse IgG HRP	Beyotime Biotechnology	1:1000
Rabbit anti-activated caspase 9	Ruiying Biological, Suzhou, China	1:450
AlexaFluor 488-conjugated donkey anti-mouse IgG	Jackson ImmunoResearch Laboratories, West Grove, PA, USA	1:1000
AlexaFluor 555-conjugated donkey anti-rabbit IgG	Jackson ImmunoResearch Laboratories	1:1000
Monoclonal anti-M13 phage	Sino Biological. INC., Beijing, China	1:2000
Biotin-conjugated monoclonal anti-C-myc (9E10)	Sigma-Aldrich, St. Louis, MO, USA	1:1000
Extravidin-HRP	Sigma-Adlrich, St.Louis, MO, USA	1:1000

### Cell culture

Human neuroblastoma SHSY5Y cells, mouse neuroblastoma neuro-2a (N2A) cells or mouse photoreceptor like 661W cells were cultured in Dulbecco’s modified Eagle’s medium (DMEM) containing 10% fetal bovine serum (Fbs). Transfection of SHSY5Y, 661W cells was performed using Lipofectamine 2000 (Invitrogen, Carlsbad, CA, USA) according to the manufacturer’s instructions.

### Protein purification

For purification of the human CAPN5 protein, 100 μl of an overnight culture of *Escherichia coli.* (BL21 strain), harboring the plasmid pET28a encoding the His-tagged CAPN5, was used to inoculate 100 mL of fresh LB-Kan broth, and shaken at 37°C for 2 h. Isopropyl β-D-1-thiogalactopyranoside was then added to a final concentration of 5 mM, and incubation was continued for a further 10 h. The Flag-tagged CAPN5 was purified using an Ni+ affinity column.

For purification of the scFvs, phagemid clones were amplified and phages were extracted as described [29]. For production of soluble ScFv proteins, 1 ml inocula of E. coli HB 2151 strain was infected with a glycerol stock of an individual phage-ScFv clone and transferred into culture flasks for expression of the scFv cassette was induced by isopropyl β-D-1-thiogalactopyranoside, which was added to give a final concentration of 1mM isopropyl β-D-1-thiogalactopyranoside. Shaking was continued overnight. ScFvs were secreted into the culture supernatant and the E. coli periplasm were harvested after osmotic shock. Supernatants were then centrifuged at 10,000×g at 4°C for 30 min and clarified by filtration through 0.22 μm filters (PALL, Port Washington, NY, USA). Finally, all clarified protein fractions (supernatant and periplasmic fraction) were pooled and passed through a Ni+ affinity column and dialyzed against PBS. Purity of the eluted soluble scFvs was evaluated by SDS–PAGE on 10% gels. The concentration of the purified scFvs was determined by the BCA technique (Beyotime).

### Selection and generation of intracellular scFv

The selection method of scFv binding to human CAPN5 was essentially as described [29]. The Tomlinson I and J libraries (Geneservice, Nottingham, United Kingdom) and recombinant CAPN5 were used for screening. Briefly, 100 μl of 22.5 μg/ml CAPN5 in PBS pH 7.4 were coated overnight at 4°C onto a 96-well tissue culture dish (Jet, biofial, Beijing, China). Wells were then blocked with 3% BSA (fatty acid free, Merck, Whitehouse Station, NJ, USA) in PBS at room temperature for 1 h. After washing the wells twice with PBS, 10^13^ phagemid particles in 0.5% BSA in PBS were added to the wells. After incubation for 40 min at room temperature, wells were washed eight times with PBS containing 0.1%, 0.3%, or 0.5% Tween-20 and then rinsed twice with PBS, for 5 min each. Bound phages in each well were released by incubation with 100 μl trypsin (Beyotime, Hai Men, China) (10 μg/ml in PBS) for 1 hour at room temperature and collected. For amplification, phages were used to infect the E. coli strain TG1. Bacteria were grown at 37°C overnight on TYE plates containing 100 μg/ml ampicillin and 1% glucose. After three rounds of panning, individual phage clones were selected for ELISA. For phage ELISA, each well of a 96-well plate was coated overnight at 4°C with 100 μl of 10 μg/ml CAPN5 in PBS, and blocked with 3% BSA in PBS for 1 hour at room temperature. Supernatants from individual clones were added to the wells, incubated at room temperature for 40 min and washed three times with PBST (PBS, 0.1% Tween 20). Wells were then incubated with a 1:3,000 dilution of the monoclonal mouse anti-M13 horseradish peroxidase (HRP) conjugated antibody (GE Healthcare) in 3% BSA in PBS for 1 hour at room temperature and washed three times with PBST. Binding of phages was detected using TMB (3, 3′, 5, 5′-tetramethylbenzidine; Beyotime). For selection of scFvs, 96 well plates were coated overnight at 4°C with 100 μl purified recombinant CAPN5 in PBS over a concentration range of 0–10 nM. Wells were blocked with 3% BSA in PBS for 1 hour at room temperature. Individual scFvs (100 μl, 100 ng/ml in PBS containing 3% BSA) were added to the wells, incubated at room temperature for 40 min, and washed with 0.1% PBST three times. Wells were then incubated with biotin-conjugated mouse anti-c-myc monoclonal antibody 9E10 for 1.5 hours at room temperature, washed three times with 0.1% PBST, and then incubated with ExtrAvidin-HRP (Sigma-Aldrich) for 1 hour. Wells were washed, and binding was detected using TMB as a substrate.

The sequences of selected clones were determined with the primer LMB (5′-CAG GAA ACA GCT ATG AC-3′) by the dideoxy chain terminating method. Sequencing was repeated three times for verification. For constructs of intracellular expressed scFvs, we subcloned c-myc and His-tagged scFvs into the pSin Puro vector from the pSin EGFP Puro plasmid using BamH I and EcoR I.

### RT-PCR

The 661W and SHSY5Y cells were transfected with CAPN5 plasmids, the cells were lysed and total RNA was extracted using TRIzol reagent (Invitrogen, USA) respectively, and cDNA was synthesized using reverse transcriptase (TIANGEN, Beijing, China). The RNA (1%) was reverse transcribed to complementary deoxyribonucleic acid, and 20 ng of complementary DNA was used as the template for RT-PCR. The amplification cycling reactions (35 cycles) were performed as follows: 2 mins at 95°C, 30 seconds at 60°C and 1 mins at 72°C. The primers’ information were used in this study (as shown in Table 2). The mRNA levels of TLR4, TLR6, IL1alpha, TNFalpha and GAPDH were determined for each experiment.

**Table 2:** Primers used for RT-PCR

Gene Name	Forward (5’-3’)	Reverse (5’-3’)	Amplicon Size (bp)	Reference Sequence
*m_TLR4*	ATGGCATGGCTTACACCACC	GAGGCCAATTTTGTCTCCACA	253	NM_021297.3
*m_TLR6*	GATCCCCGGCTCTTCGTAGAT	GACGATTACGTCCACCCACTC	196	NM_011604.3
*m_IL1alpha*	TCTATGATGCAAGCTATGGCTCA	CGGCTCTCCTTGAAGGTGA	159	NM_010554.4
*m_TNFalpha*	GAAATGCCACCTTTTGACAGTG	TGGATGCTCTCATCAGGACAG	324	NM_013693.3
*m_GAPDH*	AATGGATTTGGACGCATTGGT	TTTGCACTGGTACGTGTTGAT	157	NM_001289726.1
*h_TLR4*	AGACCTGTCCCTGAACCCTAT	CGATGGACTTCTAAACCAGCCA	127	NM_138554.4
*h_TLR6*	CGCCACTGACGACTCACTC	CTGCCACAAACCAGCAGTTG	243	NM_006068.4
*h_IL1alpha*	TGAGTGTCTCTGTTGAAAACCTC	GGGGTACTTCTATTGAACGACGA	162	NM_000575.4
*h_TNF alpha*	ATGATGGCTTATTACAGTGGCAA	GTCGGAGATTCGTAGCTGGA	231	NM_000594.3
*h_GAPDH*	TGTGGGCATCAATGGATTTGG	ACACCATGTATTCCGGGTCAAT	81	NM_002046.5

### Immunostaining

For living cells immunostaining, the living cells on coverslips were incubated with scFvs which tagged with His and C-myc at 10μg/ml in PBS for 2 hours at 37°C incubator, then discard scFvs and fixed cells with 4%PFA, incubated cells with mouse anti-his tag antibody (1:500) and goat anti-CAPN5 antibody (1:700) overnight at 4°Covernight, after PBS washing, incubated coverslips with secondary Alexa 488nm donkey anti-mouse IgG and Alexa 555nm donkey anti-goat IgG antibodies for 1 hour at room temperature. For normal immunostaining, 661W and SH-SY5Y cells were cultured for 40 hours on PDL-coated glass slides and fixed in 4% paraformaldehyde (PFA) as described [30]. After 48 hours pSin C4 scFv plasmid transfection, the cells on coverslips were fixed and incubated with mouse anti-C-myc antibody (1:500) and goat anti-CAPN5 antibody (1:700) at 4°Covernight, then following incubated with secondary Alexa 488nm donkey anti-mouse IgG and Alexa 555nm donkey anti-goat IgG antibodies for 1h at room temperature. DAPI was used to stain nuclei. Images were captured in digital format using a Zeiss microscope (Carl Zeiss, Chester, VA).

### Western blot analysis

Western blot analysis was performed as described [30]. For CAPN5 or scFvs overexpression experiments and scFv protein treatment experiments, Cells were transfected with plasmids for 60 h, or treated with scFvs at 10 μg/ml in DMEM with 0.1% FBS for 24 hours, after transfections and treatments, cells were collected and lysed. The total cell proteins were separated by SDS-PAGE and transferred to PVDF membrane, the membranes were incubated with rabbit anti-activated caspase 3, caspase 9 antibodies, goat anti-CAPN5, monoclonal anti-IL1alpha, anti-His, or anti-C-myc antibodies in 5% milk which diluted in PBS at 4°Covernight respectively, after washing with 0.1%TBST, the membrane incubated with donkey anti-mouse/goat IgG HRP secondary antibodies for 1 hour at room temperature.

For H_2_O_2_ exposure experiments, cells were pre-treated with 10 μM H2O2 for 2, 6, 12, 24 hours, then the cells were collected and lysed for immunoblot analysis as above. Bands were visualized with an enhanced chemiluminescence system kit (Beyotime). Signals were detected and quantified using Image J software (National Institutes of Health, Bethesda, MD, USA).

### Determination of cell viability

Cells were suspended in DMEM/F-12 medium to a concentration of 5 ×10^4^ cells/ml, and 100 μl was added to each well of a 96-well plate. After treatment with transfection of CAPN5 plasmids, scFvs, or normal control plasmids for 24, 48, or 60 h, cells were incubated separately with 10 μl MTT (500 μg/mL) for 4 h. The culture medium was then removed, and 100 μl dimethylsulfoxide (DMSO) was added to each well, followed by a 30 min incubation period at 25°C. Absorbance was measured spectrophotometrically at 540 nm.

### Statistics

Statistical analyses were performed with SPSS 13.0 software (IBM Corporation, Armonk, NY, USA). All data are presented as means±SEM unless otherwise specified. Student’s t-test was used for comparisons in experiments with only two groups. In experiments with more than two groups, ANOVA was performed followed by Tukey’s post hoc test for pairwise comparisons among three and greater than three groups. For analysis of more than two groups of non-parametric data, the Kruskal–Wallis test was used.

## Abbreviations

ADNIV: autosomal dominant neovascular inflammatory vitreoretinopathy
CAPN5: calpain 5calcium-activated cysteine protease
scFv: single chain fragment of variable
IL1: interlukin 1
TNF: tumor necrosis factor
TLR: toll like receptor
PBS: phosphate buffer saline
GAPDH: glyceraldehyde-3-phosphate dehydrogenase
